# Natural and Synthetic Bioactives for Skin Health, Disease and Management

**DOI:** 10.3390/nu13124383

**Published:** 2021-12-08

**Authors:** Jean Christopher Chamcheu, Anthony Lynn. Walker, Felicite Kamdem Noubissi

**Affiliations:** 1 School of Basic Pharmaceutical and Toxicological Sciences, College of Pharmacy, University of Louisiana, Monroe, LA 71209-0497, USA; 2 School of Clinical Sciences, College of Pharmacy, University of Louisiana, Monroe, LA 71209-0497, USA; 3 Department of Biology, Jackson State University, Jackson, MS 39217, USA

The skin is the largest organ of the integumentary system, strategically located at the interface of the body’s internal and external environment. It therefore serves diverse functions, including the maintenance of skin homeostasis, and provides both physically and immunologically protective barriers in view of safeguarding normal human health [1]. These functions are enshrined within the three distinct but interconnected and intercommunicative tissue compartments that make up the skin: the outermost epidermal layer, the median dermal layer and the innermost subcutaneous layer (the hypodermis). All components intercommunicate via resident cells such as keratinocytes, Langerhans cells, melanocytes and other immune cells, which secrete immune mediators such as cytokines, chemokines, extracellular matrix proteins, growth factors and hormones [2]. Healthy skin, being a major component of our physical appearance, also plays an important role in our social and sexual relationships. The perturbation of skin health, due to extrinsic or intrinsic factors, leads to or is a risk factor for several cutaneous diseases, most of which are chronic, heterogeneous, recalcitrant or incurable. The two major risk factors for skin diseases include modifiable (e.g., environmental factors such as diverse wavelengths of ultraviolet (UV) radiation types, injuries, etc.) and non-modifiable (e.g., genetics, population, epidemiological, association, etc.) factors that are associated with the pathophysiology of several dermatoses, including those that are heritable and immune-mediated, as well as skin cancers [1]. Complications in the skin may result in deleterious effects on systemic health and well-being, such as sepsis. Several abnormalities mostly affect keratinocytes, which constitute over 85% of the major epidermal cells, as seen in cancers and other inflammatory skin conditions, and a few others affect melanocytes (reviewed in [1,2]). Importantly, defects in melanocytes, the skin-pigment-producing cells, are known to cause several skin and autoimmune conditions, such as melanoma, and other pigmentation disorders, including vitiligo, melasma, etc. [1,3]. Melanogenesis is a biological process of melanosome formation and melanin (a group of pigments produced by diverse living organisms including plants, animals and microbes) biosynthesis. Melanin exerts different physiological functions, including photoprotection, the regulation of temperature, the chelation of metal ions and the quenching of free radicals [4,5]. Although melanogenesis protects melanocytes against UV-induced damage, excessive production of melanin in the skin can lead to hyperpigmentation and an array of pigmentation disorders, as well as aging. Studies have also shown that in patients with metastatic melanoma, melanotic melanomas positively correlated with a significantly lower overall survival (OS) than amelanotic melanomas [6].

Meanwhile, several nutritional and diet-derived factors provide an important contribution to the maintenance of normal skin integrity, since they can implement causative, preventive and/or therapeutic functions in several skin-associated heath conditions. Therefore, the condition of our skin can depict actual nutritional and health statuses subsequent to nutritional choices. Bioactive natural products and their synthetic derivatives have garnered tremendous interest in health promotion, disease prevention and management since the ancient medieval era. These biologically active natural phytonutrients, extracts and their synthetic derivatives have numerous potential health benefits that are poised within their regulatory properties on critical physiological processes of life, such as transcriptional and translational programs, metabolism, differentiation and growth. These natural products have demonstrated efficacy in protecting the skin against premature aging, environmental health-related risks and infection. They also ameliorate skin conditions associated with inflammatory diseases such as atopic dermatitis, psoriasis, diabetic and chronic wounds in addition to skin cancers. Appropriate in vitro and preclinical model systems of human diseases have been employed to provide a mechanistic understanding of disease pathogenesis as well as proof of concepts targeting molecular biomarkers that may be useful for therapeutic benefits.

This Special Issue is a snippet of published original research, communications and quality reviews of the scientific literature that further increase our understanding of the role of natural dietary and synthetic bioactive compounds for skin health promotion, disease prevention and management. The findings not only highlight those natural phytonutrients, extracts and synthetic scaffolds that target diverse disease molecular markers, but delineate promising strategies to reduce the burden of cutaneous human diseases and beyond.

The first paper by Michalak et al. (2021) is a literature review of the effects of bioactive micro- and macronutrients such as vitamins, minerals, fatty acids, polyphenols and carotenoids on skin heath conditions, and how healthy dietary habits could serve as useful approaches in anti-aging interventions. It highlights the effects of these compounds on skin parameters, including elasticity, firmness, wrinkles, senile dryness, hydration and color, as well as their role in the processes of skin aging [7].

The second review by Kaur et al. (2021) assessed numerous skin fungal infections, impinging on their possible treatments and the effective utilization of plant extract and oil-embedded polysaccharide-based nanohydrogels. They discuss the advantages of the use of plant extract and oil-based nanohydrogels as emerging technologies in the treatment of various skin fungal infections over the use of standard drugs that require a long treatment time with harmful side effects and often low efficacy. Plant extract-based nanohydrogels can be applied to infected wound sites as they have great penetration power, are directly absorbed through the skin, reach the hypodermis or subcutaneous layer and effectively suppress fungal infections [8]. Nutraceuticals have been proposed to have beneficial effects on many other chronic inflammatory diseases of the skin as well.

Trzeciak et al. (2020) reported that the skin barrier defects in cutaneous T-cell lymphomas (CTCLs) mirrored those observed in atopic dermatitis (AD) with regard to their expression levels of genes and proteins of the cutaneous cornified envelope (CE) in CTCL, AD and healthy skin. They identified changes in their gene and protein expressions as well as their associations with the pathogenesis of CTCL and AD. These authors identified decreased mRNA levels of FLG, FLG2, LOR, CRNN and SPRR3v1, while increased mRNA levels of RPTN, HRNR and SPRR1Av1 were observed in lesioned and non-lesioned AD skin compared to healthy control samples. In contrast, they observed increased mRNA levels of FLG, FLG2, CRNN and SPRR3v1 as well as decreased mRNA levels of RPTN, HRNR and SPRR1Av1 in CTCL skin when compared to lesioned AD skin. The study suggested that in addition to AD, these markers, FLG, FLG2, RPTN, HRNR and SPRR1A, also play a key role in skin barrier dysfunction in CTCL and thus could be considered as consistent differential diagnostic biomarkers for both AD and CTCL, thus opening up opportunities for further studies on a larger study group [9].

In a study, Adamczyk-Grochala et al. (2020) investigated the biological activity of water and ethanolic extracts of modified microalgal clones within the context of their applications in cosmeceutical and regenerative medicine. They used normal human fibroblasts and keratinocytes, and found that treatment with modified extracts of the microalga Planktochlorella nurekis variant attenuated the development of oxidative-stress-induced senescence in these human skin cells. The authors concluded that these effects may be related to increased nitric oxide, niacin and biotin levels in cells compared to an unmodified microalgal clone. Their data suggested that selected microalgal extracts of Planktochlorella nurekis could be used in an anti-skin aging regimen [10].

Another study by Gęgotek et al. (2019) used proteomics profiling of 3D keratinocytes cultures exposed to UVA or UVB radiation to identify a synergistic cytoprotective effect of rutin and ascorbic acid [11]. Because of their antioxidant and anti-inflammatory properties, combinations of ascorbic acid and rutin are often used in oral preparations and thus can be used to protect against the effects of cutaneous UV radiation from sunlight. In particular, the authors observed alterations in the expression of proteins involved in the antioxidative response, DNA repair, inflammation, apoptosis and protein biosynthesis compared to when compounds are used individually [11].

Another study by Choi et al. (2020) investigated the effect of konjac (Amorphophallus konjac) glucomannan (KGM) on damaged skin. KGM is widely used as a dietary supplement with anti-inflammatory and immunosuppressive effects, in addition to skin regenerative potential in patch or sheet form. They reported that KGM alleviates and repairs/regenerates UVB-induced skin damage by increasing the proportion of proliferative young cell populations in UVB-exposed senescent human epidermal primary melanocytes and UVB-damaged human fibroblasts in a dose-dependent manner. This was the case as they found that the mRNA and protein levels of age- and pigmentation-related factors decreased depending on the rate of new cells generated. They identified KGM as a highly effective natural agent for maintaining skin homeostasis via the promotion of a reconstituted dermal matrix against UVB-induced acute senescence or skin damage [12].

Quah et al. (2020) investigated the anti-allergic, antioxidant and anti-inflammatory activities of the ethanolic extract of Cornus officinalis (COFE) as a potential treatment of AD. Here, using RBL-2H3 cells sensitized with the dinitrophenyl-immunoglobulin E (IgE-DNP) antibody after stimulation with dinitrophenyl-human serum albumin (DNP-HSA), they observed that COFE suppressed the release of β-hexosaminidase in a concentration-dependent manner. The extract was also observed to significantly inhibit both lipopolysaccharide (LPS)-induced nitric oxide (NO) production and the expression of iNOS and pro-inflammatory cytokines (IL-1β, IL-6 and TNF-α) in RAW 264.7 cells in addition to TNF-α-induced apoptosis in HaCaT cells. Collectively, this study suggests that COFE might inhibit allergic responses, oxidative stress and inflammatory responses by a mechanism that disrupts the binding of IgE to human high-affinity IgE receptors (FceRI), and that these inhibitory effects might be achieved by COFE’s compounds loganin, cornuside and naringenin 7-O-β-D-glucoside [13].

In another study by Kim et al. (2020), the usefulness of propolis, a resinous substance generated by bees, in treating UV-induced photoaging in human skin was introduced. Propolis treatment was also found to suppress UV-induced matrix metalloproteinase (MMP)-1 production and expression in human dermal fibroblasts as well as block collagen degradation in human skin tissues, suggesting that the anti-skin-aging activity of propolis can be mimicked in clinically relevant conditions. Their investigations revealed that propolis, especially its main components caffeic acid phenethyl ester, quercetin and apigenin, shows anti-skin-aging effects through the direct suppression of phosphoinositide 3-kinase (PI3K) activity. The content of active compounds was quantified, and among the compounds identified from the propolis extract, caffeic acid phenethyl ester, quercetin and apigenin were shown to attenuate PI3K activity. These results demonstrate that propolis shows anti-skin-aging effects through the direct inhibition of PI3K activity [14].

In another study, Hyuna Lee and Eunmi Park (2021) showed that Perilla leaf and callus extracts exert a protective effect against UVB-induced damage on skin and/or keratinocytes by a mechanism that involves an increased DNA repair response and G1/S cell cycle arrest [15].

Kim et al. (2021), in a randomized, double-blind and placebo-controlled study revealed the anti-inflammatory and anti-pathogenic bacterial activities of a dietary Lactobacillus plantarum, CJLP55, a natural product supplement, on clinical improvement, skin sebum, hydration and urine bacterial EV phylum flora in patients with acne vulgaris [16].

Furukawa et al. (2021) used neonatal normal human epidermal keratinocytes (NHEK-Neo) and interleukin-10 knockout (IL-10 KO) mice to show that eggshell membrane (ESM) has a beneficial effect on maintaining skin health and improving the skin aging process. The molecular mechanism was associated with this, which upregulates calcium signaling and results in an increased expression of keratinocyte differentiation markers (including keratin 1, filaggrin and involucrin) as well as growth factors, such as transforming growth factor β1, platelet-derived growth factor-β and connective tissue growth factor. They also showed that ESM changed keratinocytes’ morphology and suppressed skin thinning [17].

Oh et al. (2021) showed that ethanolic extracts of Lithospermum erythrorhizon (LE) roots improve symptoms associated with AD (atopic dermatitis) in an NC/Nga AD mouse model. They found that LE restored defects in skin barrier function and the balance of T helper (Th) 1/Th2 AD-related cytokine and chemokine expression in the dorsal skin, serum and splenocytes of the NC/Nga mouse. This improvement of AD-related symptoms correlated with a reduction in the serum levels of IL-4, IgE and histamine via the regulation of the IgG1/IgG2a ratio, as well as the restoration of the Th1/Th2 immune balance in NC/Nga Mice. LE also decreased the levels of AD-related cytokines and chemokines in NC/Nga mouse serum and regulated the balance of cytokine and chemokine secretion in splenocytes in addition to their expression in the dorsal skin [18].

Olivera-Castillo et al. (2020) evaluated the bioactive-enriched components found in the sea cucumber (*Isostichopus badionotus*) body wall that promote anti-inflammatory activity and found that fucosylated chondroitin sulfate, the primary glycosaminoglycan, reduced the expression of critical genes, such as NF-kB, TNFa, iNOS and COX-2, and further lessened the inflammation and tissue damage caused by 12-O-tetradecanoylphorbol-13-acetate (TPA) in a mouse ear inflammation model [19].

Lee et al. (2019) demonstrated that triacylglycerol (TAG) metabolism is related to the acyl-ceramide (Cer) synthesis and corneocyte lipid envelope (CLE) formation involved in maintaining the epidermal barrier. Their investigation demonstrated that dietary borage oil-enhanced TAG content, the gene expression of TAG metabolism, acyl-Cer synthesis and CLE formation in the epidermis of essential fatty-acid-deficient guinea pigs [20].

A high melanin content has been shown to significantly reduce the efficacy of radiotherapy and chemotherapy in patients with metastatic melanotic melanomas, probably due to the radioprotective and free radical scavenging properties of melanin. Therefore, inhibiting melanogenesis has been suggested as a single, combination or adjuvant approach in the treatment of melanotic melanomas.

This Special Issue has also highlighted the utility of natural products tested and has shown their inhibitory properties in melanogenesis as well as their contribution to the treatment of melanotic melanoma or pigmentation disorders affecting the skin. These natural products include extracts of lotus seedpod (LSE), sorghum bicolor, black cumin seed and lactobacillus-helveticus-fermented milk whey (LHMW).

Hsu et al. (2020) showed that lotus seedpod extract (LSE), or its main active compound epigallocatechin (EGC), downregulates the signaling pathway, leading to melanin production in the α-MSH-induced murine melanoma cell line B16F0 by inhibiting the expression of MC1R, the expression of tyrosinase and the expression of microphtalmia-associated transcription factor (MITF). These authors also detected a reduction in the activity of p38 and protein kinase A (PKA) in the cells treated with LSE. These results were supported by their in vivo studies that showed a similar downregulation of melanin synthesis in mice ears treated with LSE or ECG and exposed to UVB. They suggest that the mechanism by which LSE inhibits melanin synthesis involves the inhibition of p38 and PKA signaling pathways [21].

Recently, Han et al. (2020) demonstrated the anti-melanogenic role of ethanolic extract of sorghum bicolor (ESB) in B16/F10 cells treated with the melanin synthesis inducer 3-isobutyl-1-methylxanthine (IBMX). The authors showed that IBMX-induced melanogenesis was drastically reduced in the presence of ESB by a mechanism involving the inhibition of MITF, tyrosinase and tyrosinase-related protein 1. ESB contains phenolic compounds such as 9-hydroxyoctadecadienoic acid (9-HODE), 1,3-O-dicaeoylglycerol and tricin, and exhibits a high antioxidant property. It could be used as a skin-whitening material in cosmetics or in the management of melanocytic melanomas [22].

Similarly, Ikarashi et al. (2020) showed that lactobacillus-helveticus-fermented milk whey (LHMW) suppressed melanin synthesis in B16 mouse melanoma treated with α-MSH by inhibiting MITF, tyrosinase and tyrosinase-related protein 1 at the RNA and protein levels [23].

Li et al. (2020) found that the black cumin seed extract Thymocid also inhibits melanin synthesis in B16F10 cells by a mechanism that plausibly involves the downregulation of MITF and tyrosinase activity. In addition, Thymocid treatment reduced collagenase and elastase activities as well as protein glycation and collagen cross-linking. This portrays Thymocid as a potential anti-aging compound [24].

Overall, the 18 papers published in this Special Issue highlight that an adequate dietary intake of natural products or their application is essential for maintaining normal skin homeostasis, thereby being crucial for human health. In summary, the daily dietary intake of natural products such as fruits and vegetables is a cost-effective and healthy way to strengthen human skin, significantly reducing the risk of skin disorders and improving general well-being. These natural dietary products are potential drugs for the management of premature aging and hyperpigmentation as they can serve as whitening agents or as chemopreventive and chemotherapeutics, as well as an adjuvant in the treatment of melanotic melanomas or other skin disorders involving melanogenesis and other chronic skin disorders. However, additional studies using more physiologically relevant models are warranted to determine their efficacy and potential clinical applications. Validating the beneficial effects of these natural and synthetic bioactives would improve the management of skin health and reduce the burden associated with the plethora of emerging skin diseases.
